# Application of the propensity score in a covariate-based linkage analysis of the Collaborative Study on the Genetics of Alcoholism

**DOI:** 10.1186/1471-2156-6-S1-S33

**Published:** 2005-12-30

**Authors:** Betty Q Doan, Constantine E Frangakis, Yin Y Shugart, Joan E Bailey-Wilson

**Affiliations:** 1 Department of Epidemiology, Johns Hopkins Bloomberg School of Public Health, Baltimore, Maryland, USA, 21205; 2 Statistical Genetics Section, Inherited Disease Research Branch, National Human Genome Research Institute, National Institutes of Health, Baltimore, Maryland, USA, 21224; 3 Department of Biostatistics, Johns Hopkins Bloomberg School of Public Health, Baltimore, Maryland, USA, 21205

## Abstract

**Background:**

Covariate-based linkage analyses using a conditional logistic model as implemented in LODPAL can increase the power to detect linkage by minimizing disease heterogeneity. However, each additional covariate analyzed will increase the degrees of freedom for the linkage test, and therefore can also increase the type I error rate. Use of a propensity score (PS) has been shown to improve consistently the statistical power to detect linkage in simulation studies. Defined as the conditional probability of being affected given the observed covariate data, the PS collapses multiple covariates into a single variable. This study evaluates the performance of the PS to detect linkage evidence in a genome-wide linkage analysis of microsatellite marker data from the Collaborative Study on the Genetics of Alcoholism. Analytical methods included nonparametric linkage analysis without covariates, with one covariate at a time including multiple PS definitions, and with multiple covariates simultaneously that corresponded to the PS definitions. Several definitions of the PS were calculated, each with increasing number of covariates up to a maximum of five. To account for the potential inflation in the type I error rates, permutation based *p*-values were calculated.

**Results:**

Results suggest that the use of individual covariates may not necessarily increase the power to detect linkage. However the use of a PS can lead to an increase when compared to using all covariates simultaneously. Specifically, PS3, which combines age at interview, sex, and smoking status, resulted in the greatest number of significant markers identified. All methods consistently identified several chromosomal regions as significant, including loci on chromosome 2, 6, 7, and 12.

**Conclusion:**

These results suggest that the use of a propensity score can increase the power to detect linkage for a complex disease such as alcoholism, especially when multiple important covariates can be used to predict risk and thereby minimize linkage heterogeneity. However, because the PS is calculated as a conditional probability of being affected, it does require the presence of observed covariate data on both affected and unaffected individuals, which may not always be available in real data sets.

## Background

Alcohol dependence has been shown to cluster in families. Multiple linkage analyses have been performed for phenotypes related to alcoholism, identifying phenotype-specific linkage evidence [1-5]. To increase the statistical power to detect linkage in the presence of heterogeneity, we explored the use of covariate-based linkage analysis based on a conditional logistic regression model [6,7]. Because one degree of freedom is added to the statistical test for each additional covariate analyzed, we incorporated a propensity score (PS) to collapse multiple covariates into one variable and showed in simulation studies it consistently improved the statistical power of the linkage test [[8,9], unpublished data, 2004]. Rosenbaum and Rubin [10] first described the PS in a causal inference analysis to control for multiple covariate effects that could potentially bias assessments of treatment effect outcomes when randomization experiments were not possible. In such a setting, the score is defined as the conditional probability of being assigned to a treatment group given the covariate data, and in practice, it can be estimated from the observed covariate data with a logistic model of the treatment group assignment based on the covariates. The PS used here is instead defined as the conditional probability of being affected given the observed covariate data in families, and its predicted value is then used as the single covariate in Olson's conditional logistic regression model [6]. A covariate-based linkage analysis on the Collaborative Study on the Genetics of Alcoholism (COGA) microsatellite dataset was performed.

## Methods

### Study population and data collection

The study population consisted of families ascertained by the COGA. The COGA study and the data available for the Genetic Analysis Workshop 14 (GAW14) have been previously described in this issue. This study specifically uses the microsatellite genotype and covariate data that were released as part of GAW14.

### Genome-wide linkage analysis using covariates

Covariate-based affected relative pair linkage analysis using single-point identity-by-descent (IBD) probabilities and a general conditional logistic model was performed as implemented in GENIBD and LODPAL of S.A.G.E. v4.6 [6,7,11] on the microsatellite genotype data across the entire genome. In LODPAL, all affected relative pairs are treated as independent observations, and a single covariate value is calculated for each affected relative pair as the sum of the covariate values for the two affected relatives in the pair. The trait selected was ALDX1, defined as alcoholism based on both the DSM-III-R [12] and the Feighner criteria [13]. Covariates considered were age at interview (age_int), sex, maximum number of drinks (maxdrinks), smoking status (smoker), and ttth1, an electrophysiological measurement of brain activity. Additionally five different propensity scores were defined, and their corresponding regression coefficients are listed in Table 2. LOD scores were calculated by incorporating into the analysis no covariates, each covariate alone (including the different single covariate PS definitions), and all covariates simultaneously.

These PS values were derived from a logistic regression of affection status on the covariate data, using the model described below:

 where x_j _= the j^th ^covariate

The affection status was coded as 1 for affected and 0 for unaffected. This logistic regression was performed in STATA (v8.2) [14] on the entire dataset, and the predicted value of the probability of affection for each individual was used as the individual's PS corresponding to the set of observed underlying covariates. The presence of measured covariates on both affected and unaffected individuals is required, although only affected individuals are used in the linkage analysis itself.

### Determination of significance and comparison of linkage evidence across analysis methods

Significance was determined by permutation testing. Affection status coupled with its covariate values was permuted within families generating 1,000 replicates, and single-point linkage analysis was performed on the observed data and on each replicate. The *p*-value of a test statistic was calculated as the proportion of permutations whose statistic was equal to or greater than the observed value. Two types of statistics were computed. The first was a LOD score for each marker. The second was the sum of LOD scores across all markers, selected to simultaneously capture multiple regions of significant linkage evidence. Because the statistics were compared to their reference permutation distribution in the calculation of the *p*-values for each method, the relative proportion of significant tests between methods is an indication of relative power.

## Results and Discussion

Table 1 displays the overall *p*-value and the number of significant markers across the genome according to the analysis method used (set of covariates analyzed). Including individual covariates did not necessarily lead to more significant loci identified as linked, and could even result in fewer significant findings of linkage compared to analyses with no covariates. However including a propensity score (such as with PS1 and PS3) can greatly increase the number of significant linkage results. Additionally, including the PS (except for PS2) did result in more significant regions of possible linkage compared with its corresponding multiple covariates method.

These results suggest that incorporating multiple covariates together may be more productive than the use of individual covariates in linkage analysis of complex diseases. Specifically, age at interview, sex, and smoking status (PS3) appear to be important covariates that can be used to account for heterogeneity associated with alcoholism. The inclusion of PS3 in the linkage analysis led to both the most significant overall *p*-value as well as the largest number of different markers yielding some significant evidence for linkage. The most significant individual markers were GATA193 (*p *= 0.0044) on chromosome 17, D2S200, D6S477, and D15S644 (all three with *p *= 0.0078). In the logistic regression for PS3 (Table 2), the smoking variable resulted in the greatest odds ratios (OR of 5.33 ± 1.02) among any of the covariates used in defining a PS, and for PS1, the sex variable resulted in one of the lowest ORs (0.108 ± 0.019) of any covariates. For PS2 and PS5, which identified the smallest number of markers with significant linkage, approximately one-third of the ttth1 covariate data was missing. Thus, examining the values of the regression coefficients in the calculation of the PS and the goodness of fit of the logistic models may be a means to help define the most appropriate PS.

**Figure 1:**
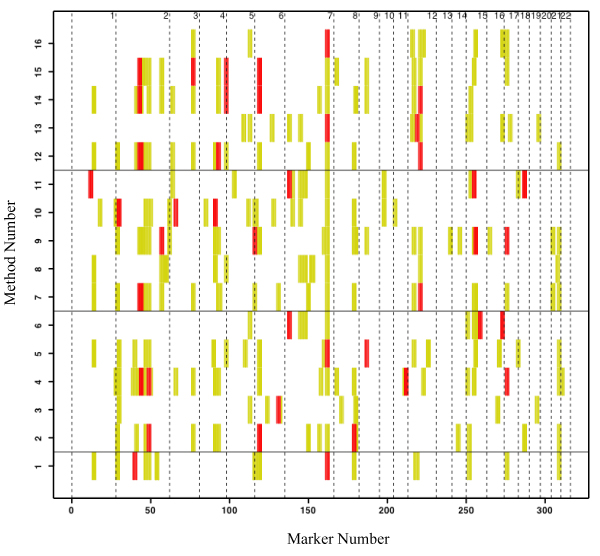
**Markers with significant linkage evidence according to the method of analysis. **The yellow bars represent markers significant at the 0.05 level, and the red bars represent markers significant at the 0.01 level. The marker numbers correspond to 315 microsatellite markers throughout the genome, and are separated into chromosomes 1 through 22. The method number corresponds to the set of covariates analyzed as listed in Table 1, and is separated by the type of method used. From bottom to top, the methods are no covariates, single covariates, propensity scores, and multiple covariates.

To examine whether the markers yielding significant evidence for linkage were consistent across the methods, Figure 1 displays a plot of the markers with LOD scores at the significance level of 0.05 (yellow) and 0.01 (red) for each analysis method. From bottom to top, the first level represents no covariates analyzed, the second level represents the methods with individual covariates analyzed, the third level represents all the PS methods, and the fourth level represents linkage analysis with the multiple covariates. Across these groups, several regions consistently provided significant linkage evidence regardless of the method of analysis, as defined as having at least eight methods resulting in significant evidence. These regions correspond to markers (with number of methods in parentheses) on: chromosome 2 (#47 D2S1790 (9), #48 D2S1331 (7), and #49 D2S373 (10)), chromosome 6 (#119 D6S1018 (8)), chromosome 7 (#162 D7S509 (15)), and chromosome 12 (#221 D12S1090 (8)). Evidence on chromosome 7 has been widely replicated in multiple studies [1-4]. However, the location of the red bars representing the most significant markers was not consistent across methods. It should be noted that with 1,000 permutations performed, the lowest empirical *p*-value that can be attained is *p *< 0.001, representing the situation in which none of replicate LOD scores was more extreme than the observed LOD score.

## Conclusion

The incorporation of covariate information into a linkage analysis can potentially increase the power to detect linkage by identifying more loci with linkage evidence and also increased statistical linkage evidence for identified loci. Because the addition of each covariate into the analysis inflates the type I error rate in this likelihood model, it is important to use empirically derived *p*-values to determine significance. Having corrected for the inflation in the type I error rate, the use of a propensity score (except for PS2) compared with the use of all the covariate simultaneously does lead to the identification of more linked loci in this study. Even though several regions of significant linkage were consistent across the analysis methods, the location of the most significant regions was not consistent. Thus it is also important to emphasize that despite the power increase, the selection of covariates to include into the analysis method must be done carefully and the identification of the significant linkage regions can vary based on the covariates used. However, defining a PS that results in the covariates having the largest OR away from the null may be a means to identify important covariates for the PS, and the use of that PS may result in the greatest overall power gain to detect linkage.

## Abbreviations

COGA: Collaborative Study on the Genetics of Alcoholism

GAW14: Genetic Analysis Workshop 14

IBD: Identity by descent

OR: Odds ratio

PS: Propensity score

## Authors' contributions

All authors participated in the discussions of the study design and statistical methodology and helped draft the manuscript. BQD performed the statistical analysis and CEF assisted with programming. All authors have read and approved the final manuscript.

## Figures and Tables

**Table 1:** Overall *p*-values and the number of significant microsatellite markers by analysis method

Method No.	No. cov.	Covariates analyzed	Overall *p*-value^b^	No. markers with *p*-values:	
				
				<0.05	<0.01
Single covariate					
1	0	none	0.001	16	2
2	1	age_int	0.002	18	3
3	1	sex	0.838	9	1
4	1	maxdrinks	0.002	27	4
5	1	smoker	0.008	19	2
6	1	ttth1	0.055	10	3

Propensity scores^a^					
7	1	PS1 (age_int, sex)	0.002	22	3
8	1	PS2 (age_int, sex, ttth1)	0.046	12	0
9	1	PS3 (age_int, sex, smoker)	<0.001	27	4
10	1	PS4 (age_int, sex, smoker, maxdrinks)	0.008	21	3
11	1	PS5 (age_int, sex, smoker, maxdrinks, ttth1)	0.329	14	4

Multiple covariates					
12	2	age_int, sex	0.025	19	4
13	3	age_int, sex, ttth1	1.000	16	2
14	3	age_int, sex, smoker	0.003	18	4
15	4	age_int, sex, smoker, maxdrinks	0.045	16	4
16	5	age_int, sex, smoker, maxdrinks, ttth1	1.000	9	1

Methods were identified according to the set of covariates analyzed.

^a ^PS were calculated from the covariates listed, and the corresponding regression coefficients are listed as odds ratios (OR) with the standard errors (SE) in Table 2.

^b^The overall *p*-value for the analysis method was calculated as the probability of obtaining the observed sum of the LOD scores across the genome in the 1,000 permuted replicates. Significance thresholds of <0.05 and <0.01 were used.

**Table 2:** Regression coefficients as odds ratio (SE) for the five propensity scores used

No. cov.	Propensity score definitions (included covariates)	Regression coefficents as OR (SE)				
		
		age_int	sex	smoker	max drinks	ttth1
1	PS1 (age_int, sex)	**0.955**^a^**(0.005)**	**0.108 (0.020)**	**-**	**-**	**-**
1	PS2 (age_int, sex, ttth1)	**0.947 (0.008)**	**0.093 (0.023)**	**-**	**-**	0.871 (0.150)
1	PS3 (age_int, sex, smoker)	**0.947 (0.006)**	**0.137 (0.026)**	**5.328 (1.025)**	**-**	**-**
1	PS4 (age_int, sex, smoker, maxdrinks)	0.973 (0.010)	1.696 (0.622)	**2.643 (0.901)**	**1.709 (0.080)**	**-**
1	PS5 (age_int, sex, smoker, maxdrinks, ttth1)	0.969 (0.019)	2.039 (1.139)	2.320 (1.216)	**2.149 (0.204)**	1.026 (0.362)

^a^The significant coefficients (*p*-value < 0.05) are in bold.
